# Perceiving speech from a familiar speaker engages the person identity network

**DOI:** 10.1371/journal.pone.0322927

**Published:** 2025-05-14

**Authors:** Gaël Cordero, Jazmin R. Paredes-Paredes, Katharina von Kriegstein, Begoña Díaz

**Affiliations:** 1 Department of Psychology, Faculty of Medicine and Health Sciences, Universitat Internacional de Catalunya, Barcelona, Spain; 2 Faculty of Psychology, Technische Universität Dresden, Dresden, Germany; University of Hamburg, GERMANY

## Abstract

Numerous studies show that speaker familiarity influences speech perception. Here, we investigated the brain regions and their changes in functional connectivity involved in the use of person-specific information during speech perception. We employed functional magnetic resonance imaging to study changes in functional connectivity and Blood-Oxygenation-Level-Dependent (BOLD) responses associated with speaker familiarity in human adults while they performed a speech perception task. Twenty-seven right-handed participants performed the speech task before and after being familiarized with the voice and numerous autobiographical details of one of the speakers featured in the task. We found that speech perception from a familiar speaker was associated with BOLD activity changes in regions of the person identity network: the right temporal pole, a voice-sensitive region, and the right supramarginal gyrus, a region sensitive to speaker-specific aspects of speech sound productions. A speech-sensitive region located in the left superior temporal gyrus also exhibited sensitivity to speaker familiarity during speech perception. Lastly, speaker familiarity increased connectivity strength between the right temporal pole and the right superior frontal gyrus, a region associated with verbal working memory. Our findings unveil that speaker familiarity engages the person identity network during speech perception, extending the neural basis of speech processing beyond the canonical language network.

## Introduction

Each human voice is acoustically unique, a feature which allows us to recognize familiar speakers, but which adds computational complexity to speech perception, i.e., the process by which speech sounds are decoded from the continuous speech signal and identified. Voice and linguistic information are intertwined in the speech signal to an extent that the acoustic cues that identify speech sounds (i.e., phonemes) vary across speakers [1]. For instance, the percept of ambiguous vowels changes in accordance with the acoustic properties of voices [2,3]. Prior experience with a voice increases the robustness of the neural representation of speech and facilitates speech intelligibility [4–6]. Remarkably, the brain can harness voice cues with minimal exposure. In experimental contexts, the effects of voice priors on speech perception and recognition have been observed after just a few minutes or as little as two sentences of exposure to the speaker’s voice [7–10]. The use of voice priors in speech perception is in line with proposals of human perception which argue that the central nervous system forms stable percepts of highly variable stimuli, including phonemes, by exploiting its cumulative knowledge [11,12].

Interactions between voice and speech processes are unexpected when considering the different brain regions involved. Voice-sensitive regions (i.e., responsive to speaker information embedded in the speech signal) are primarily right-lateralized and are functional constituents of the person identity network [13,14]. This network includes regions that exhibit unimodal sensitivity, such as the voice-sensitive anterior temporal lobe and superior temporal sulcus, as well as regions that have been proposed to integrate information associated with person identities, such as the supramarginal and angular gyri [13–22]. Speech perception predominantly engages the left superior temporal sulcus and gyrus [23], which in turn are part of the language network. This network also includes the bilateral left inferior and middle frontal gyri, and middle temporal gyrus [23–26]. Previous neuroimaging studies have identified two potential neurofunctional mechanisms which might support the use of voice priors during speech perception. Firstly, studies have reported an increase in interhemispheric functional connectivity between right voice-sensitive regions and left speech-sensitive regions when recognizing speech from multiple speakers as opposed to recognizing speech from a single speaker [27–29]. Secondly, several studies have identified an overlap between the neural substrates of voice perception and recognition and speech perception; areas along the temporal cortices and right temporoparietal junction are sensitive to both voice and phonetic information [5,21,22,29–31]. Most of the studies that have found evidence in favor of either of these two neurofunctional mechanisms investigated how physical properties of the speaker’s voice, such as pitch or vocal tract length, modulated brain responses during the performance of speech tasks. To the best of our knowledge, only one study has investigated how the brain exploits voice priors during a speech recognition task. Holmes and Johnsrude (2021) studied the brain responses associated with speech recognition from familiar and unfamiliar speakers in two listening conditions: in the presence of competing speech and in the absence of competing speech. Responses in left posterior temporal regions, not including the primary auditory cortex, displayed greater similarity between listening conditions for the familiar speakers as compared to the unfamiliar ones. Holmes and Johnsrude interpreted these findings as an indication that speech representations are more resistant to competing speech when the target speaker is familiar. Their finding suggests that voice familiarity interacts with goal-driven attention to facilitate speech recognition in noisy environments [5]. In the present study we investigate whether functionally overlapping regions are also engaged when perceiving speech from a familiar speaker in the absence of competing speech, and therefore when attention to voice properties is not task relevant.

**Fig 1:**
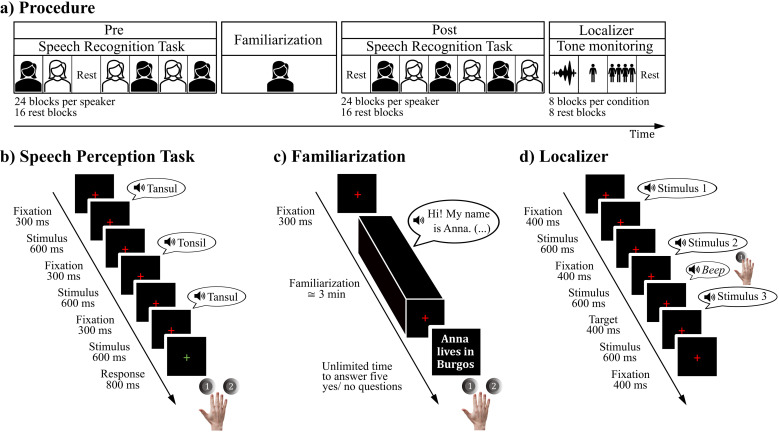
Procedure and trial design of the different tasks that the participants conducted in the scanner. (a) Shows the four runs that composed the procedure. The first three runs (i.e., Pre, Speaker Familiarization, and Post) corresponded to the experiment while the fourth run corresponded to the Independent Functional Localizer for voice and speech-sensitive regions. (b) Depicts the trial structure of the speech perception task that participants conducted in the Pre and Post runs. Each block was composed of four trials. Trials followed an ABX design; participants had to respond if the third stimulus of each trial was a repetition of either the first or second stimulus presented during that trial. (c) Portrays the run in which participants were familiarized with one of the speakers. It began with the presentation of a recording of one of the speakers featured in the speech perception task. This recording was a first-person narration which contained autobiographical information of a fictional character. When this recording finalized, five statements concerning the identity of the fictional character were presented. Participants had to judge whether these affirmations were true or false. Note that, despite the text of the Familiarization appearing in the English language in the figure, the Familiarization was conducted in the native language of the participants, Spanish. (d) Portrays the three conditions that composed the Independent Functional Localizer for voice and speech-sensitive regions: spectrally rotated speech, single-speaker, and multi-speaker. During the Independent Functional Localizer participants were instructed to press a button when a pure tone was presented.

We hypothesized that perceiving speech from a familiar speaker, relative to an unfamiliar speaker, would engage the neural mechanisms proposed to support the use of voice-specific information during speech perception: interhemispheric connectivity [27–29] and functionally overlapping regions [5,21,22,29–31]. Twenty-seven adults were included in this functional magnetic resonance imaging (fMRI) study. We measured Blood-Oxygen-Level-Dependent (BOLD) activity during a speech perception task with auditory disyllabic non-words before and after familiarizing participants with one of the speakers featured in the task (Fig 1). An independent functional localizer was employed to define voice-sensitive and speech-sensitive Regions of Interest (ROI), allowing us to test for changes in connectivity and BOLD activity in these specific regions, as observed in previous studies [5,21,22,27–31]. Drawing from the two neurofunctional mechanisms that have been proposed to support the interaction between voice and speech processes, we expected that the voice sensitive region would exhibit an increase in interhemispheric connectivity in association with conducting the speech perception task with the familiar speaker, relative to the unfamiliar speaker. Furthermore, we predicted that speaker familiarity, relative to speaker unfamiliarity, would be associated with greater responses in regions in which voice and speech processes exhibit neural overlap, such as the right posterior superior temporal gyrus [21] or left posterior middle temporal gyrus [30].

## Methods

### Participants

A total of 31 graduate and undergraduate students were recruited for this study between the 20^th^ of January 2022 and the 24^th^ of January 2024. Technical errors related to the MRI-compatible headphones and the response box rendered the fMRI data of three participants unsuitable for analysis. Moreover, the behavioral data of one participant suggested that random responses had been provided throughout the speech perception task (Pre accuracy: 48.95%; Post accuracy: 48.43%). These four participants were excluded from all analyses. The final sample consisted of 27 adults (15 female) whose mean age was 21 years, ranging from 18 to 26. This sample size is similar than that of recent studies which have investigated interactions between voice and speech processes by means of fMRI [5,21]. All participants were right-handed according to the Oldfield handedness questionnaire [32]. Participants were native Spanish speakers who did not have substantial musical training, since musicianship has been associated with enhanced voice processing abilities [33]. Substantial musical training was defined as meeting a minimum of 2 of the 3 following criteria; (1) the onset of musical training having occurred prior to the age of 12 years, (2) having partaken in musical training for a minimum of 5 years, and (3) being part of a musical group or ensemble, either currently or in the past (criteria as in [34]). None of the participants had a history of auditory, neurological, psychiatric, language or learning disorders, and they had normal or corrected-to-normal vision. The Ethics Committee of the Medical Faculty and Health Sciences of the Universitat Internacional de Catalunya (Spain) approved the procedures (Study: PSI-2020-05; version: 17/11/20). All participants provided their written informed consent and were monetarily rewarded for their time (10€/ hour).

### Stimuli

For the experiment, two sets of 40 disyllabic non-words were created. One set was built with Spanish phonemes and the other with Arab phonemes (each set was designed by a native speaker of the respective language). The purpose of the two language datasets will be reported elsewhere. Two adult (19 and 24 years) female Arab-Spanish native bilinguals recorded the stimuli, amounting to a total of 160 non-words. The two speakers also recorded a text describing autobiographical details of a fictional character in the first-person, henceforth referred to as the Familiarization, which had a duration of 2 minutes 45 seconds (see Appendix 1 for the original text in Spanish and an English translation). Previous studies have shown that directing the attention of listeners to identity characteristics of a speaker leads to voice-specific characteristics being learnt [21,35,36] as well as improving speech intelligibility [9]. Thus, we designed the Familiarization with the intent of familiarizing participants with both the identity and with voice-specific characteristics of the familiar speaker. Five written statements concerning the contents of the Familiarization were also created (see S1 Table in Appendix 1 for the original statements in Spanish and an English translation).

Three native Spanish listeners (including two of the authors) considered that the identity of the speakers was prone to confusion on account of the speakers having relatively similar-sounding voices. To remedy this, two versions of each speaker -a tone higher and a tone lower than the original voice- were created by changing the pitch of the original recordings (“Change Pitch”, Audacity v. 3.0.2, Audacity Team). The manipulated versions were employed in the experiment. Manipulation of the pitch has been successfully used to create different perceptual versions of a single voice [29,37,38]. The original, unmodified stimuli were not used.

A third set of 40 disyllabic non-words with Spanish phonemes was assembled for an independent functional localizer of voice and speech-sensitive areas. Four native Spanish speakers, different from the speakers from the experiment, recorded the non-words. As with the stimuli from the experiment, we increased the acoustic diversity of the voices by creating a tone higher and a tone lower version of each voice (“Change Pitch” function, Audacity v. 3.0.2, Audacity Team). All 12 voices were used in the localizer. A copy of the resulting 480 stimuli (40 non-words x 12 voices) were spectrally rotated, a process which preserves the spectral complexity of speech while removing all linguistic information [39], by means of a custom script (Blesser3, Version 3.1., downloaded from: https://www.phon.ucl.ac.uk/resource/software-other.php) in MATLAB (Version R2022b, MathWorks, Inc., Natick, MA USA). By employing different stimuli in the independent functional localizer from those employed in the experimental procedure, we sought to ensure that the results obtained from the analysis of our experimental data are specific to the experimental conditions and not confounded by the characteristics of the stimuli employed during the experimental procedure [40]. Multiple speakers were recorded and a copy of the stimuli was spectrally rotated due to the characteristics of our independent functional localizer protocols, which followed previous studies [39,41]. For further details regarding the independent functional localizer protocols, please see the Intependent functional localizer section).

Stimuli recordings were conducted using an Audio-Technica AT2020 microphone, a Marantz Professional Sound Shield Live vocal reflection filter, and with the software Audacity (Version 3.0.0., Audacity Team) in a sound-attenuated room. A noise reduction procedure was applied to all audio clips with Audacity software. The volume of all audio clips was normalized to 75 dB, a fade was applied to the first and last 50 ms of all clips, and the duration of all non-words was equalized to 600 ms with the PRAAT Vocal Toolkit [42] (“stretch” method available in the “Change Duration” function).

### Procedure

The fMRI procedure (Fig 1, panel a) consisted of two parts: the experiment and an independent functional localizer to identify the location of the voice and speech-sensitive regions in our group of participants. To avoid our results being influenced by cognitive effort, we designed all tasks with the intent of high accuracy being easily attainable in all conditions. Participants were familiarized with the experimental procedure before entering the MRI-machine. They performed 8 trials of the experimental task with one of the speakers employed in the independent functional localizer. Both the experiment and the independent functional localizer were performed using a custom Presentation script (Version 22.1, Neurobehavioral Systems, Inc., Berkeley, CA). Audiovisual stimuli presentation was controlled using a VisuaStim Digital system (Resonance Technology Inc., Northridge, CA). Auditory stimuli were delivered through MRI-compatible Serene Sound headphones (Resonance Technology Inc., Northridge, CA) which offer an attenuation of 30 dB of scanner noise. Visual stimuli were presented via MRI-compatible goggles also manufactured by Resonance Technology Inc.

#### Experiment.

The experimental paradigm aimed at evaluating the effect of the factor speaker familiarity (i.e., Familiar (Fam) and Unfamiliar (Unfam)) on speech perception. During two runs (i.e., Pre and Post) participants performed a speech perception task which followed an ABX design. Three non-words were sequentially presented in each trial. The first and second non-words differed, while the third was a repetition of either the first or the second. Participants were tasked with responding if the third non-word was a repetition of the first or of the second non-word presented in the trial. Throughout the experiment, non-words were enunciated by two speakers (i.e., Fam and Unfam), with only one speaker per trial and block (Fig 1, panel b). The two runs, Pre and Post, followed a block design with the same procedures. Thus, the experiment contained four condition blocks: Fam/Pre, Unfam/Pre, Fam/Post, and Unfam/Post. Between the Pre and the Post, the Familiarization of one of the two speakers was presented (Fig 1, panel c). Despite our use of the Fam – Unfam nomenclature in the Pre run, it is crucial to note that during the Pre run, participants had no prior experience with neither speaker. Only in the Post run was a familiar speaker presented, since the Familiarization occurred after the Pre run. However, for the sake of simplicity, we will employ the nomenclature Fam/ Unfam to distinguish speakers regardless of run. In other words, we refer to the speaker with whom participants were familiarized with as the Fam speaker in both the Pre and Post run, despite participants not being familiar with said speaker during the Pre run.

Each run (i.e., Pre and Post) contained 24 blocks per speaker (i.e., Fam and Unfam). Each block was composed of 4 trials. A trial began with the presentation of a red cross. After a 300 ms delay, three auditory non-words were presented with an inter-stimulus interval (ISI) of 300 ms. The first and second non-words were different from one another, and the third non-word was a repetition of either the first or the second. Participants performed an ABX task: they had to indicate which of the two initial non-words was repeated in the third place by pushing with their right index or middle finger one of two buttons on a response box. Participants were instructed to press the button positioned under their index finger to indicate that the repeated non-word was the first non-word presented during the trial, while pressing the button assigned to their middle finger indicated that the repeated non-word was a repetition of the second. Response time started from the beginning of the third non-word until 800 ms after its offset, as indicated by the change of the red fixation cross to green. Each block had a duration of 14 seconds and each trial of 3 and a half seconds. Sixteen rest blocks, which also had a duration of 14 seconds, were also presented. During the rest blocks, no auditory stimulation was presented, and a red fixation cross appeared on the screen. The 4 block conditions (i.e., Pre/Fam, Pre/Unfam, Post/Fam, and Post/Unfam) were pseudo-randomly presented, with the restriction of not allowing the consecutive presentation of more than five blocks of the same condition. The selection of the stimuli that composed each condition block was restricted to avoid repeating non-words between trials of a single block. Each non-word was enunciated by one of the two speakers in each run to diminish perceptual learning effects related to the use of the same speaker - non-word pair in the two runs. The presentation of the rest blocks was also pseudo-random, with two rest blocks never being presented consecutively.

Between the Pre and Post runs, participants were informed that one of the two speakers was going to introduce themselves and that they would later be tested on autobiographical details associated with the identity of the speaker. Participants then heard the Familiarization, a ca. 3 minute speech sample narrated in the first-person that contained extensive autobiographical information, from one of the two speakers employed in the Pre and Post runs. Participants were informed prior to the familiarization that they would be tested on the autobiographic details associated with the identity of the speaker, encouraging them to attend the presentation. The Familiarization was followed by 5 visually presented statements concerning the autobiographical information of the fictional character. Participants had to judge whether these statements were true and provided their answers without the pressure of a time limit by pushing one of two buttons with either their right index or middle finger. Pressing the button positioned under their index finger indicated that the participant judged the affirmation to be true, while pressing the middle finger button indicated that they considered the affirmation to be false. The speaker that acted as the familiar speaker and the version (i.e., high or low pitch) was counterbalanced across participants to ensure that potential differences between the familiar and unfamiliar speakers were not stimuli driven. The unfamiliar speaker had the opposite pitch dimension (either high or low) than the familiar speaker to ensure discriminability. The duration of the experimental procedure was approximately 35 minutes (Pre and Post = 15 minutes each; Familiarization = circa 5 minutes).

#### Independent functional localizer.

The independent functional localizer for voice and speech-sensitive regions was administered after the experiment (Fig 1, panel a). The localizer followed a block design with three different conditions: single-speaker, multi-speaker, and spectrally rotated speech (Fig 1, panel d). Non-words were presented in the single-speaker and multi-speaker condition blocks, while spectrally rotated speech was presented in the equally named condition blocks. Throughout the localizer, participants conducted a pure-tone detection task.

The independent functional localizer was composed of 8 blocks per condition. All condition blocks had a duration of 14 seconds and consisted in the presentation of 14 auditory stimuli with an ISI of 400 ms while a red fixation cross was presented. The design of the three condition blocks is based on previous studies [39,41]. In the single-speaker blocks, participants heard 14 non-words enunciated by a single voice. The voice employed in each single-speaker block was randomly selected and the non-words were pseudo-randomly determined to avoid repeating the same non-word more than once per block. In the multi-speaker blocks, participants listened to one non-word enunciated by 14 voices. For each block, the non-word was selected randomly, and the voices were pseudo-randomly ordered to avoid consecutive presentations of the same voice. In the spectrally rotated speech blocks, participants listened to 14 spectrally rotated non-words enunciated by a single voice. The speaker was randomly determined for each block and the spectrally rotated non-words were pseudo-randomly selected to avoid within-block repetitions.

Twice per condition block, a 200 ms pure tone (1300 Hz) was presented following a 200 ms delay after the last syllable of a block. The blocks in which the pure tone was presented were pseudo-randomly determined to avoid the presentation of the tone in adjacent blocks. Participants indicated that they had heard the pure tone via button press with their right index finger. Responses were considered correct if delivered up to 1 and a half seconds after the onset of the pure tone. Eight rest blocks of the same duration as the condition blocks, i.e., 14 seconds, were also presented. No auditory stimulus was presented during the rest blocks, solely a red fixation cross. The presentation of condition and rest blocks was pseudo-randomized to avoid the consecutive presentation of two blocks of the same condition or of two rest blocks. The duration of the independent functional localizer was 7m 30s.

#### Data acquisition.

Functional images and structural T1-weighted images were acquired on a Siemens 3T Magnetom Prisma Fit MR scanner with a 20-channel head coil (Siemens Healthcare, Erlangen, Germany) at the Hospital Clínic in Barcelona (Spain). Adjustable padding was placed on both sides of the participants’ heads to stabilize head position. For the functional images, we used a Multi-Band Echo-Planar Imaging (MB-EPI) sequence (slice thickness = 2.6 mm, number of slices = 42, order of acquisition = interleaved, multiband acceleration factor = 6, TR = 875 ms, TE = 30 ms, flip angle = 65°, FOV = 250 mm, voxel size = 2.6 mm^3^; phase encoding direction: anterior-posterior). We employed a slice tilt of -30° to reduce signal losses in the temporal lobes [43,44], where previous studies have localized voice and speech-sensitive regions [13,14,24–26,45]. A total of 1024 EPI volumes were acquired during both the Pre and the Post runs (192 volumes per condition and 128 rest volumes per run). A total of 512 volumes (128 volumes per condition and 128 rest volumes) were acquired during the independent functional localizer. Additionally, a B0 field-map was acquired before the first functional run (Short TE = 4.92 ms, Long TE = 7.38 ms). Lastly, we acquired the T1-weighted structural images by using a high-resolution GRAPPA EPI sequence (slice thickness = 0.80 mm, number of slices = 208, GRAPPA factor = 2, TR = 2400 ms, TE = 2.22 ms, FOV = 256 mm).

### Data analysis

All participants included in the data analysis: (i) achieved a response accuracy above 75% in the speech perception task in both runs of the experimental procedure, (ii) provided correct answers to a minimum of four of the five Familiarization questions, and (iii) provided an answer to a minimum of five of the six presentations of the pure tone during the independent functional localizer. Behavioral response accuracy in the experimental task was analyzed with a generalized linear mixed effects model fitted in R (Version 4.1.0, R Core Team, 2017) with the addition of RStudio (Version 2022.2.2.485, RStudio Team) and the lme4 package [46]. We conducted a trial-by-trial analysis in which response accuracy was modelled as the dependent variable, Run (2 levels: Pre and Post), Speaker (2 levels: Fam and Unfam), and the interaction of Run and Speaker as fixed effects, and participant ID as a random effect with random intercept. We also conducted trial-by-trial analysis of the reaction time data with a linear mixed effects model which had the same structure as the generalized mixed effects model employed to analyze the accuracy data. Behavioral effects were considered significant at p < .05. The fMRI data was preprocessed and analyzed with SPM 12 (Wellcome Centre for Human Neuroimaging, London, UK; implemented in MATLAB Version R2022b, MathWorks, Inc., Natick, MA USA) with the addition of the CONN toolbox [47,48].

#### Preprocessing of the fMRI data.

For each participant, the preprocessing pipeline began with realigning and unwarping the fMRI data. During unwarping, the B0 field-map was used for susceptibility distortion correction. Preprocessing continued with the identification of potential outlier scans from the global BOLD signal and the estimation of motion parameters of each subject during image acquisition. Following standard procedures [47,48], acquisitions which exhibited framewise displacement above 0.9 mm or global BOLD signal changes above 5 standard deviation were flagged as potential outliers and were considered as confounding effects during the denoising procedure conducted prior to the functional connectivity analyses. Functional images were then co-registered to the participant’s anatomical image and normalized to Montreal Neurological Institute (MNI) standard stereotactic space. Functional data was smoothed with an 8mm full width half maximum Gaussian kernel.

For the functional connectivity analyses, the preprocessed functional data was denoised using the CONN Toolbox. Confounding effects to the BOLD signal (noise components from cerebral white matter and cerebrospinal fluid, estimated motion parameters, identified outlier scans, and constant task effects) were used as temporal covariates and removed from the BOLD functional data by linear regression. The BOLD time series was then bandpass filtered between 0.008 and 0.09 Hz to reduce the effect of low-frequency drifts and high-frequency physiological noise [49].

#### Functional connectivity analysis.

We sought to identify changes in functional connectivity associated with speaker familiarity during speech perception. Hence, our seed region for all connectivity analysis was the voice-sensitive Region of Interest (ROI) (see Independent functional localizer section) which is known to respond to the experimental manipulation conducted here, i.e., voice familiarity [13,16,17,45,50–56]. We used the CONN Toolbox to perform both seed-to-whole brain and ROI-to-ROI analyses using the implemented generalized Psychophysiological Interaction (gPPI) procedure [57,58]. In the seed-to-whole brain analysis, target voxels were all voxels in the brain, except for those contained in the seed region. For the ROI-to-ROI analysis, target voxels were solely those voxels included in the speech-sensitive ROI (see Independent functional localizer section). To conduct gPPI, we first extracted an averaged BOLD time-course of the seed region and used it as a physiological regressor. For the first-level analysis, we generated a PPI regressor for each condition (i.e., Pre/Fam; Pre/Unfam; Post/Fam; Post/Unfam) by calculating the element-by-element product between psychological and physiological regressors. We then computed how strongly the time course of the seed region correlated with the PPI regressor of a target voxel. This pair-wise computation was made for every possible seed-target pair to measure task-dependent changes in functional connectivity for each participant. These results were converted to z-scores using the Fisher’s z-transformation before calculating group-level averaged functional connectivity scores. An interaction contrast [(Post/Fam – Pre/Fam) – (Post/Unfam – Pre/Unfam)] was computed to investigate if the functional connectivity of the Voice-Sensitive ROI (VSR) is modulated by speaker familiarity during speech perception. To discard the possibility that differences in functional connectivity were caused by behavioral performance differences, we performed a second analysis with the interaction contrast in which we included as control covariates the accuracy and reaction time associated with the speech perception task. For the accuracy covariate, the accuracy percentage participants attained in each experimental condition were transformed into rationalized arcsine units to increase the data’s suitability for statistical analysis [59]. The reaction time covariate was included in milliseconds. The accuracy and reaction time covariates of each participant were both calculated following the interaction contrast computed for the neuroimaging data analysis: (Post/Fam – Pre/Fam) – (Post/Unfam – Pre/Unfam). Moreover, we conducted exploratory analyses to investigate if the behavioral covariates exhibited a significant correlation with modulations in functional connectivity. For completeness, main effect contrasts of Run [(Post/Fam + Post/Unfam) – (Pre/Fam + Pre/Unfam)] and of Speaker [(Post/Fam + Pre/Fam) – (Post/Unfam + Pre/Unfam)] were also conducted.

#### Activity analysis.

The activity analysis aimed to investigate changes in brain responses associated with speaker familiarity during speech perception. Activity analysis was conducted on the preprocessed data prior to denoising. Statistical parametric maps were generated for each participant by modeling the evoked hemodynamic response of each block type separately (i.e., Pre/Fam; Pre/Unfam; Post/Fam; Post/Unfam) as boxcar functions convolved with a synthetic hemodynamic response function using the general linear model approach [60]. Following a similar approach as in the functional connectivity analyses, we conducted an interaction contrast as well as contrasts modelling both main effects (i.e., Run and Speaker) at the first level. At the second level, one-sample t-tests across the first-level contrasts images of all participants were used. To ascertain that the results of our main analysis (i.e., the interaction contrast) were not due to differences in participant’s performance in the speech perception task, we conducted a second analysis in which the accuracy and reaction time scores of participants were included as control covariates (see Functional connectivity analysis section for details on the calculation of the behavioral scores included as covariates). As in the connectivity analyses, exploratory analyses were conducted to investigate if the behavioral covariates exhibited a significant correlation with modulations in BOLD activity.

#### Statistical thresholds.

For the connectivity analysis, results were considered significant at a voxel-height (cluster-forming) threshold of *p* < .001 and a cluster size threshold at *p* < .05 corrected for False Discovery Rate (FDR), in accordance with the recommended thresholds for cluster-level inferences based on Random Field Theory detailed in the handbook of the CONN toolbox [47]. The Harvard-Oxford cortical atlas, as implemented in the CONN toolbox, was employed to identify the regions in which significant results were obtained. Activity analyses effects were considered significant if they were present at p < .05 Family Wise Error (FWE) corrected at the peak level and a minimum cluster size of 10 voxels at the whole-brain level, or at p < .05 FWE corrected at the peak level for the ROI and Bonferroni corrected for the two ROIs (p < .025 FWE corrected). Regions in which significant activity results were obtained were labelled using the Neuromorphometrics atlas implemented in SPM12.

#### Definition of regions of interest (ROIs).

We used the independent functional localizer to define group-based ROIs. For each participant, we computed a statistical parametric map by modelling the evoked hemodynamic response for the three conditions separately (single speaker, multi-speaker, and spectrally rotated speaker) as boxcar functions convolved with a synthetic hemodynamic response function using a general linear model approach [60]. To define the VSR we followed an approach similar to that employed by Belin & Zatorre (2003) [41]. We contrasted the BOLD response elicited by the multi-speaker condition (i.e., varied voice information; constant phoneme information) against the single speaker condition (i.e., constant voice information; varied phonetic information) at the first-level and employed a one-sample t-test across the first-level contrast images of all participants at the second-level. To define the Speech-Sensitive ROI (SSR) we employed an approach presented by Scott and colleagues (2000) [39]; the BOLD responses elicited during the single speaker condition (i.e., constant voice information; varied phoneme information) were contrasted against the perception of acoustic stimuli with comparable temporal and spectral complexity as speech (i.e., spectrally rotated speech) at the first-level and a one-sample t-test across the first-level contrast images of all participants at the second-level.

The ROIs were defined as all contiguous voxels responsive at *p* < 0.05 uncorrected located in an anatomical position in line with the literature (S2 and S3 Tables in Appendix 2 for a comparison between the ROIs defined here with clusters reported by previous studies). Anatomical position was determined using the Neuromorphometrics atlas implemented in SPM12. A cluster situated in the right temporal pole which extended into the right anterior superior temporal sulcus was defined as the Voice-Sensitive Region (VSR, MNI coordinates: 42, 14, -23; k = 24 voxels) (S1 Fig, panel A in Appendix 2). The Speech-Sensitive Region (SSR) was defined as a cluster situated in the left posterior superior temporal gyrus (MNI coordinates: -57, -31, 10; k = 33 voxels) (S1 Fig, panel B in Appendix 2). The ROIs were created and exported to the functional image space using the Marsbar Toolbox [61].

## Results

### Behavioral results

Participants exhibited high accuracy in the Speech Perception Task in all the conditions that composed the experimental procedure (see Table 1). A generalized linear mixed effects model was used to examine the effect that Run (2 levels: Pre and Post), Speaker (2 levels: Fam and Unfam), and the interaction of these two factors had on the probability that participants would deliver a correct response on a trial-by-trial basis. The delivered responses (coded as correct: 1; incorrect or miss: 0) were modelled as the dependent variable, run (Pre and Post), Speaker (Fam and Unfam), and their interaction as fixed effects, and participant ID as a random effect. The estimates of the fixed effects suggested that neither Run, Speaker, nor their interaction had a significant effect on the probability of participants providing a correct response (Run: *β* = −0.15, SE = 0.13, z = −1.12, p = .26; Speaker: *β*  = 0, SE = 0.14, z = 0, p = 1; Run x Speaker: *β* = −0.027, SE = −0.19, z = −13, p = .89). Similarly, no significant effect was obtained for Run, Speaker, nor their interaction in the RT model (Run: *β* = −3.58, SE = 57.97, t = −0.06, p = .95; Speaker: *β* = −43.18, SE = 57.90, t = −0.74, p = 0.45; Run x Speaker: *β* = 32.96, SE = 81.95, t = 0.40, p = .68). The homogenous performance across experimental conditions attained by the participants suggests that any modulations in brain functional connectivity or activity associated with perceiving speech from the familiar speaker are unlikely to be caused by differences in cognitive effort between the experimental conditions.

**Table 1:** Performance in the speech perception task.

Condition	Mean	SD	Range
Accuracy (%)			
Pre/Fam	95.23	5.08	78.12–100
Pre/Unfam	95.20	3.76	85.41–100
Post/Fam	95.90	4.66	83.33–100
Post/Unfam	96.05	3.47	88.54–100
Reaction time (ms)			
Pre/Fam	768.88	132.75	410.47–988.85
Pre/Unfam	767.04	139.00	383.34–1033.05
Post/Fam	770.20	106.57	606.17–993.00
Post/Unfam	762.21	110.58	563.13–1000.73

### Functional connectivity results

**Fig 2:**
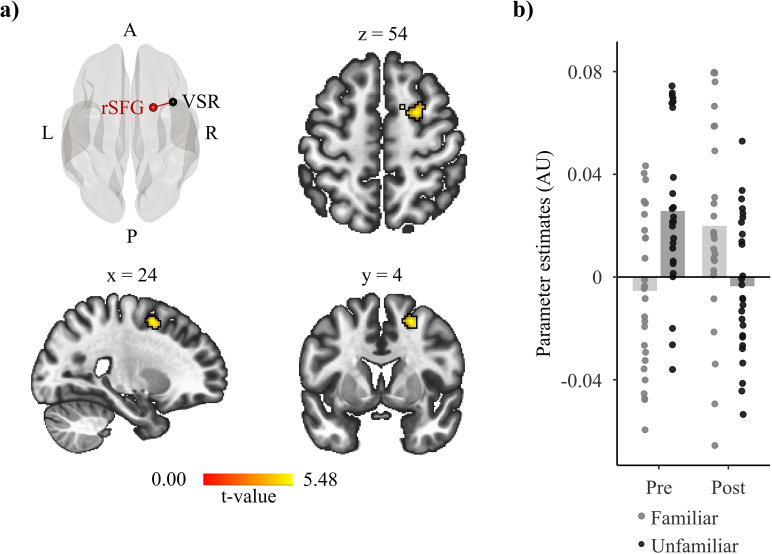
Functional connectivity results of the interaction contrast. (a) Speech perception from a familiar speaker led to a change in functional connectivity strength between the independently localized Voice-Sensitive Region (VSR), employed as seed region, and the right Superior Frontal Gyrus (rSFG; t = 5.48; p = .004 FDR-corrected). Glass brain shows the approximate location of the voxel that exhibited the strongest change in functional connectivity strength of the rSFG and VSR. Functional connectivity results were considered significant at a voxel-height threshold of p < .001 and cluster-wise threshold of p < .05 FDR-corrected. A = anterior. R = right, P = posterior, L = left. (b) Bar-plots (AU: Arbitrary Units) display the functional connectivity strength between the VSR and rSFG in each condition. Participant-specific values for each condition are represented by the scatterplots overlayed with the respective conditions. Values plotted in bar plots were extracted with the REX toolbox [62] as implemented in the CONN toolbox.

Conducting seed-to-whole brain analysis with the interaction contrast [i.e., (Post/Fam – Pre/Fam) – (Post/Unfam – Pre/Unfam)] revealed a significant effect in the connectivity of the VSR with the right Superior Frontal Gyrus (SFG; see Table 2 and Fig 2; for a comparison between the coordinates of this cluster and those of previous similar studies, see S4 Table in Appendix 4). This result cannot be explained by difficulty differences in the conditions of the speech perception task. Analysis of the behavioral data showed that task difficulty was comparable across conditions (see Behavioral results section). Furthermore, a second analysis in which the accuracy scores and RT data were added as second-level control covariates still showed significant modulations of the functional connectivity between the VSR and the rSFG (t(24) = 5.86, p = .010 FDR corrected). Exploratory analysis conducted to investigate if the behavioral covariables correlated with changes in functional connectivity strength in the interaction contrast did not reveal significant effects. The contrast modelling the main effect of Run [i.e., (Post/Fam + Post/Unfam) – (Pre/Fam + Pre/Unfam)] unveiled an increase in functional connectivity between the VSR and both the right Supramarginal Gyrus (rSMG) and the left Central Opercular Cortex. Furthermore, the main effect of Run also revealed a decrease in connectivity strength between the VSR and the right FP (see Table 2 and S3 Fig Appendix 3). The contrast that modelled a main effect of Speaker [i.e., (Post/Fam + Pre/Fam) – (Post/Unfam + Pre/Unfam)] revealed an increase in connectivity strength between the VSR and the left SFG (see Table 2 and S4 Fig in Appendix 3).

**Table 2:** Functional connectivity results of the seed-to-whole brain analyses.

Contrast ^a^	Brain region ^b^	Peak MNI coordinates			Size *p-*FDR	*k*
		*x*	*y*	*z*		
Interaction contrast: (Post/Fam – Pre/Fam) – (Post/Unfam – Pre/Unfam)	Right Superior Frontal Gyrus	24	04	54	.004	76
Main effect of Run: (Post/Fam + Post/Unfam) – (Pre/Fam + Pre/Unfam)	Right Supramarginal Gyrus (posterior division)	38	−34	28	.007	80
	Left Central Opercular Cortex	−50	−6	6	.010	64
	Right Frontal Pole	16	28	38	.017	52
Main effect of Speaker: (Pre/Fam + Post/Fam) – (Pre/Unfam + Post/Unfam)	Left Superior Frontal Gyrus	−18	18	66	.013	77

*k*, cluster size.

^a^Seed region in all contrasts was an independently localized voice-sensitive region (peak MNI coordinate: 42, 14, -23; right temporal pole).

^b^Cluster labels obtained from the Harvard-Oxford cortical atlas as implemented in the CONN toolbox.

The ROI-to-ROI analyses revealed no significant effects between the VSR and the SSR for neither the interaction contrast (T(26) = 1.19; *p*-FDR = .30) nor for the contrasts modelling the main effects of Run (T(26) =.70; *p*-FDR = .66) and Speaker (T(26) =.13; *p*-FDR = .96).

### Activity results

**Fig 3:**
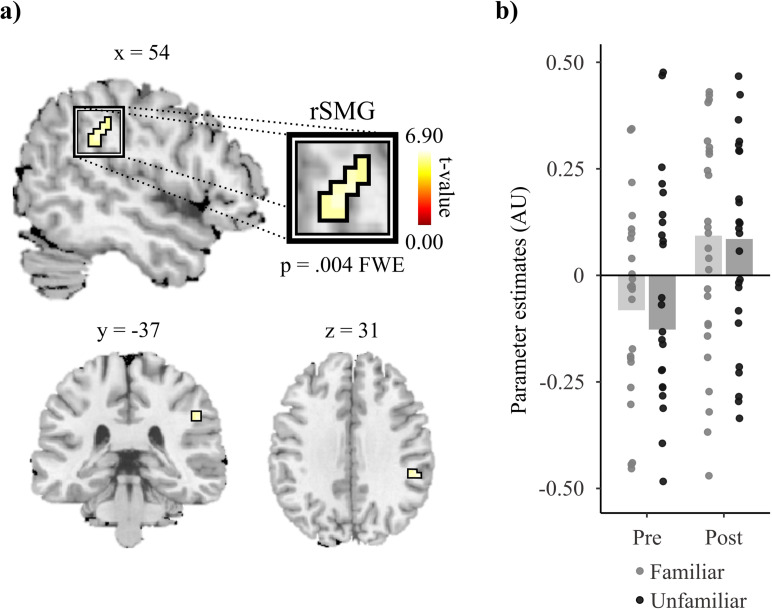
Activity results at the whole brain level. (a) The BOLD activity recorded from the right Supramarginal Gyrus (rSMG) exhibited a significant interaction for speaker familiarity and run (t = 6.90; p = .004 FWE corrected at the whole brain level). (b) Bar plots show the parameter estimates obtained from the peak voxel in each condition. Scatterplots overlayed with the respective conditions show participant-specific values. At the group level, speaker familiarity led to BOLD activity in this region increasing 0.17 AU in Post relative to Pre (i.e., Post/Fam – Pre/Fam)”.

Performing the activity analysis at the whole brain level with the interaction contrast [i.e., (Post/Fam – Pre/Fam) – (Post/Unfam – Pre/Unfam)] revealed a significant effect in the rSMG (see Table 3 and Fig 3; for a comparison between these peak coordinates and those of previous studies, see S5 Table in Appendix 4). Inspection of the first-level results revealed that 18 participants exhibited the interaction for Run and Speaker in the cluster that was significant at the second level. The sensitivity exhibited by the rSMG to speaker familiarity cannot be explained by differential degrees of difficulty in the conditions of the speech perception task; accuracy and reaction time was comparable across conditions (see section 3.1.). Furthermore, a second analysis which included these behavioral measures as second-level control covariates confirmed that the rSMG is sensitive to speaker familiarity (t(24) = 6.86; p = .007 FWE corrected). The main effect analysis of Run [i.e., (Post/Fam + Post/Unfam) – (Pre/Fam + Pre/Unfam)] revealed greater activity in two clusters which bilaterally covered the Inferior Frontal Gyri (IFGs), and a third cluster located in the right SPL (see Table 3 and S7 Fig in Appendix 3). The analysis modelling the main effect of speaker [i.e., (Post/Fam + Pre/Fam) – (Post/Unfam + Pre/Unfam)] did not reveal any significant modulation in activity.

**Table 3:** Activity results at the whole brain level.

Contrast	Brain region^a^	Peak MNI coordinates			t	Peak *p-*FWE	*k*
		*x*	*y*	*z*			
Interaction contrast: (Post/Fam – Pre/Fam) – (Post/Unfam – Pre/Unfam)	Right Supramarginal Gyrus	54	−37	31	6.90	.004	19
Main effect of Run: (Post/Fam + Post/Unfam) – (Pre/Fam + Pre/Unfam)	Left Inferior Frontal Gyrus	−45	5	19	6.92	.004	16
	Right Inferior Frontal Gyrus	39	8	25	6.83	.005	20
	Right Superior Parietal Lobule	21	−67	43	6.54	.010	10
Main effect of Speaker: (Pre/Fam + Post/Fam) – (Pre/Unfam + Post/Unfam)						n.s.	

n.s., non-significant.

^a^Regions labelled in accordance with the Neuromorphometrics atlas implemented in SPM12

**Fig 4:**
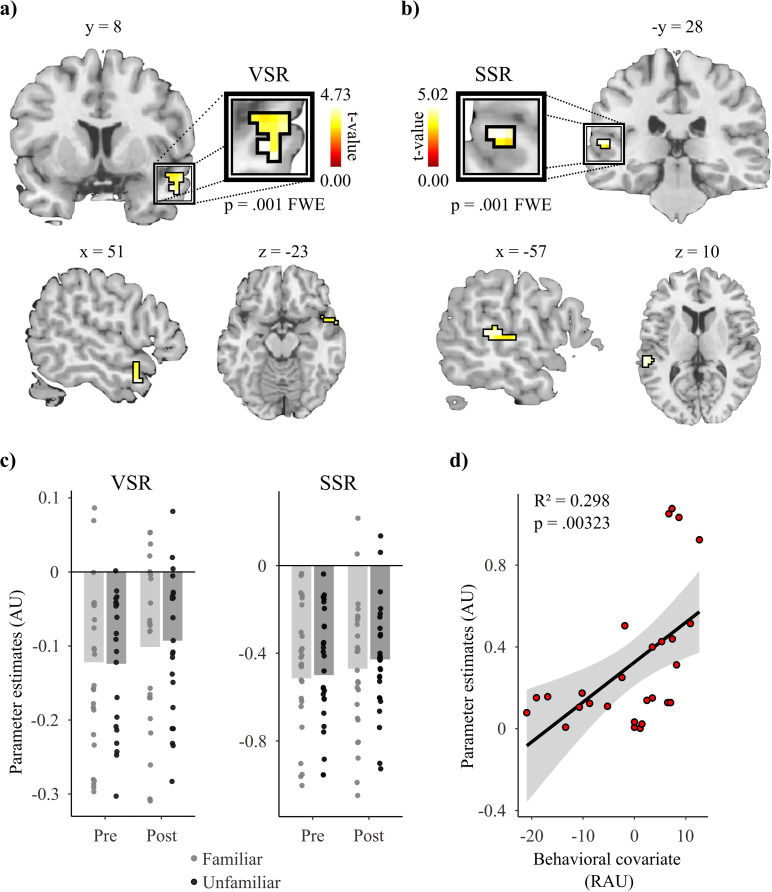
Activity results of the interaction contrast in the independently localized regions of interest. Small volume correction of the interaction contrast revealed significant interactions in both independently localized regions of interest: the Voice-Sensitive Region (VSR), located in the right temporal pole (a), and in the Speech-Sensitive Region (SSR), located in the left posterior superior temporal sulcus (b). Both results were significant (VSR: t = 4.73, p = .001, FWE corrected; SSR: t = 5.02; p = .001, FWE corrected) at p < .05 FWE corrected at the peak level for the ROI and Bonferroni corrected for the two ROIs (i.e., p < .025 FWE corrected). Bar plots (c) show the parameter estimate obtained from the peak voxel of each ROI in each condition at the group level. Scatterplots overlayed with the bar plots show participant-specific values for the respective conditions. At the group level, speaker familiarity (i.e., Post/Fam – Pre/Fam) increased the BOLD activity in the VSR and the SSR by .02 and .04 AU, respectively. (d) Exploratory analysis revealed an association between the accuracy participants attained in the speech recognition task (RAU: Rationalized Arcsine Units) and the interaction contrast parameter estimate extracted from the peal voxel of the SSR. This association was marginally significant after Bonferroni correction for the two ROIs (t = 3.36; p = .025 FWE corrected).

Employing the two independently localized ROIs for small volume correction in the interaction contrast revealed significant effects (see Fig 4) in both the VSR (MNI: 51 8–23; t = 4.73; p = .001 FWE corrected, and Bonferroni corrected for the two ROIs) and in the SSR (MNI: -57–28 10; t = 5.02; p = .001 FWE corrected and Bonferroni corrected for the two ROIs). Examination of the first-level results revealed that 27 and 24 participants exhibited the interaction effect in the VSR and SSR, respectively. The sensitivity of these two regions to speaker familiarity was confirmed by a second analysis in which the accuracy and RT scores of participants were included in the interaction contrast as second-level control covariables: a significant interaction remained present in both ROIs (VSR: t(24) = 4.60; p = .001 FWE corrected and Bonferroni corrected; SSR: t(24) = 5.80, p = .001 FWE corrected and Bonferroni corrected for the two ROIs). Lastly, exploratory analysis revealed a marginally significant association between the accuracy covariate and the interaction effect in the SSR (t = 3.36, p = .025 FWE and Bonferroni corrected).

## Discussion

There are three main findings from our study on the influence of speaker familiarity on speech perception. Firstly, speaker familiarity was associated with changes in BOLD activity in regions of the person identity network: the right Temporal Pole (rTP), which we independently localized as a voice sensitive region, and the right Supramarginal Gyrus (rSMG). Secondly, the posterior left Superior Temporal Gyrus (lSTG), a region which we localized as speech-sensitive, also exhibited sensitivity to speaker-familiarity. Thirdly, speaker familiarity led to an increase in connectivity strength between the rTP and the right Superior Frontal Gyrus (rSFG). These findings cannot be explained by difficulty differences in the conditions of the speech perception task. The accuracy and reaction time of participants was comparable across conditions and all results remained significant when analyses included these two behavioral measures as second-level control covariates. Our results align with our hypothesis of speech perception from familiar speakers engaging regions which exhibit a functional overlap between voice and speech processes. However, contrary to our predictions, perceiving speech from a familiar speaker did not lead to an increase in interhemispheric connectivity. Our findings suggest that speech perception from a familiar speaker engages regions of the person identity network, speech-selective regions which are additionally sensitive to speaker familiarity, and a right-lateralized network which, we propose, supports the harnessing of voice priors during speech perception for the encoding and maintenance of speech information in the verbal memory system.

One of our main findings is the sensitivity to speaker familiarity during speech perception exhibited by the rTP and the rSMG. Both regions have been associated with contributing to person identity recognition (rTP: 51,56; rSMG: [15,63]). However, research suggests that the specific functional contributions of these two regions to the process of speaker recognition during speech perception differ. The rTP has been proposed as being the region where person identity information is stored, allowing for the recognition of a speaker from the perception of their voice [13,16,17,41,64]. Here, the task participants conducted while their BOLD activity was recorded did not require speaker recognition. However, research has shown that speaker information is processed during speech perception despite voice properties not being task relevant [65–67]. Therefore, the sensitivity to speaker familiarity exhibited by the rTP in the present study could be attributed to the role of this region in recognizing the voice of the familiarized speaker. Regarding the rSMG, in addition to its implication in person identity recognition [15,63], research suggests that it is also involved in phoneme perception. Vaden and collaborators (2010) found that the BOLD activity of the rSMG is subject to fMRI phoneme repetition-suppression effects, which led them to propose that this region is sensitive to phoneme predictability [68]. Furthermore, evidence suggests that the rSMG supports voice information influencing phoneme perception. Myers & Theodore (2017) found that the rSMG responded to speakers producing phonemes that aligned with the listener’s prior experience of the talker’s speech. These results were interpreted as indicating the rSMG contributes to the speech perceptual system accommodating the idiosyncratic ways in which different talkers produce their phonemes [21,22]. Building on these proposals, we suggest that the sensitivity to speaker familiarity exhibited by the rSMG in the current study may reflect a similar role: monitoring whether the familiar speaker produces the expected speaker-specific variations. This monitorization would allow the speech perceptual system to detect when expectations differ from percepts. Our proposal aligns with claims of the perceptual system harnessing all available information to optimize speech perception [11,12,69–73].

Our second main finding is the sensitivity to speaker familiarity exhibited by the lSTG, a speech-sensitive region [23–26]. This finding is in line with previous studies that have reported that the lSTG is also sensitive to speaker-specific information [5,29,31]. Holmes and Johnsrude (2021) investigated speech recognition from both familiar and unfamiliar speakers under two listening conditions: one with competing speech and one without. BOLD response patterns in the posterior lSTG exhibited greater similarity between listening conditions when the target speaker was familiar, compared to when the target speaker was unfamiliar. The between condition similarity in response patterns for familiar speakers positively correlated with the benefit participants exhibited in speech comprehension from the familiar speakers. The authors interpreted their findings as an indication that top-down attention mechanisms exhibited greater engagement when processing speech from familiar speakers as compared to unfamiliar speakers in the presence of competing speech. They proposed that this increased engagement led to more robust phoneme representations in the posterior lSTG [5]. Here, we found that the BOLD activity of the posterior lSTG exhibits differential engagement during speech perception from speakers that differ in familiarity. Furthermore, our analysis revealed a marginally significant positive association between the benefit in speech perception associated with speaker familiarity and the BOLD activity interaction in the lSTG. Our findings align with the proposal by Holmes and Johnsrude (2021) of speaker familiarity leading to enhanced phoneme representations in this speech-sensitive region. However, the task design employed in the present study requires us to propose an alternative cognitive mechanism to the top-down attentional modulation proposed by Holmes & Johnsrude (2021). Our speech perception task did not feature competing speech. Therefore, directing attention to the voice properties of speakers was not task-relevant. We suggest that the enhanced phoneme representations associated with speaker familiarity in the lSTG is due to this region also encoding speaker-specific characteristics, as proposed by previous studies [29,31]. This interpretation might seem in conflict with our independent functional localization of the posterior lSTG as a speech-sensitive region. However, what we suggest is that voice information, in addition to phoneme information, is also represented in the posterior lSTG. While left lateralized temporal regions are firmly established as favoring the encodement of linguistic information [23–26], a growing body of research suggests that left lateralized temporal regions are also implicated in vocal identity processing [29,30,74–76]. This redundancy in voice information representation would allow for the robust processing of speaker-specific phonetic variations despite functional disruption of voice-sensitive regions, as a recent study has shown [77].

Our third finding corresponds to an increase in functional connectivity strength in response to speaker familiarity between the independently defined voice-sensitive region and the rSFG. The recruitment of the rSFG might be associated with the involvement of this region in verbal working memory, i.e., the cognitive ability that allows us to retain and mentally manipulate linguistic information [78–81]. The speech perception task, conducted with both the unfamiliar and familiar speaker, required the engagement of verbal working memory; participants had to retain the presented non-words to identify which non-word was later repeated. The observed increase in connectivity strength between the voice-sensitive region and rSFG in association with the familiar speaker suggests that familiarity with the voice of the familiar speaker contributed to retaining in working memory the non-words produced by said speaker during the speech perception task. Previous studies show that familiarity with stimuli improves performance in working memory tasks [82–86]. For instance, familiar faces are easier to remember than unfamiliar faces [84]. While in the present study no behavioral benefit was observed for the familiar speaker, probably due to a ceiling effect observed in all conditions of the speech perception task, previous studies show that working memory automatically recruits prior knowledge to enhance its functioning. The increase in functional connectivity between the voice sensitive area and the SFG might reflect the recruitment of voice priors for use in verbal working memory.

The three main findings of the study partially support our hypothesis. We hypothesized that perceiving speech from a familiar speaker would engage similar neurofunctional mechanisms as those observed in previous studies that have investigated the processing of voice characteristics during speech perception. Two such mechanisms have been proposed: i) interhemispheric functional connectivity between right voice-sensitive regions and left speech-sensitive regions [27–29] and ii) overlap between the neural substrates supporting both processes [5,21,22,29–31]. Our findings reveal that recognizing speech from a familiar speaker engages the second of these two mechanisms; regions which are sensitive to both voice and speech information (i.e., rSMG, and lSTG). Regarding why we did not find evidence for the other proposed mechanism; studies that have reported increases in interhemispheric connectivity during speech perception featured multi-speaker conditions [27–29], which our design did not include. These differences in design and results suggests that the engagement of interhemispheric connectivity to support speech perception is associated with scenarios which feature multiple, unfamiliar, and rapidly changing speakers. This type of multi-speaker situations are presumably more perceptually demanding relative to the presentation of speakers in isolation, as in the present study.

In addition to the findings involving interactions between speaker conditions before and after the Familiarization, our analyses revealed results associated with a main effect of Run. These results include an interhemispheric increase in connectivity strength between the rTP and the left central operculum, a region involved in auditory and linguistic processing [87], and an increase in BOLD activity in three regions involved in person identity recognition: the bilateral Inferior Frontal Gyri (IFG) and the right Superior Parietal Lobule (SPL) (for a review, see 13). The bilateral IFG are involved in voice-identity recognition [13,88–90] while both the right IFG and SPL are multimodal regions which participate in recognizing person identities that have been recently learnt in laboratory experiments [13,56,91–94]. The observed main effect of Run suggests that participants learnt voice-specific characteristics of both the familiar and the unfamiliar speakers, regardless of the availability of autobiographic information associated with the identity of each speaker. This interpretation is in line with previous neuroimaging studies that showed that greater familiarity with a voice leads to these regions of the person identity network exhibiting greater BOLD responses [52,56,90,95]. During the Pre run, participants heard each speaker enunciate non-words for approximately 3 minutes. These 3 minutes of exposure may have been enough for participants to recognize the speakers featured in the Post run as being the speakers of the Pre run, as previous studies have shown that voices can be accurately recognized after as little as a couple of sentences of exposure [9,96,97]. We suggest that the main effect of Run reflects general familiarity with the two voices featured in the task, whereas the interaction effects reflect richer, speaker-specific knowledge influencing speech perception. In support of the distinct nature of the main effects of run and the interaction effects, behavioral research has shown that the exposure required for mere voice recognition is considerably less than the exposure required for voice-related processes to influence speech perception [9]. However, it should be noted that the results of the main effect of Run could reflect task-learning effects not associated with voice information. It remains for future research to investigate whether initial exposure to a speaker activates regions such as the bilateral IFGs, with greater exposure subsequently engaging regions sensitive to both voice and speech information, as observed in the present study.

The present study reveals the engagement of regions pertaining to the person identity network, beyond voice-specific areas, during the performance of a speech perception task. However, our design does not allow us to fully attribute functional specificity to the reported changes in BOLD activity and functional connectivity as a function of speaker familiarity. While we recorded the BOLD signal of participants as they performed the speech perception task, the findings could reflect voice familiarity or voice-related processes. The absence of an experimental condition in which speech perception was not performed prevents us from unequivocally determining the nature of the connectivity changes captured in the present study. However, a recent high powered (n = 218) fMRI study found that the neural underpinnings that support the general perception of human vocalizations (i.e., speech, laughter, sighing, crying, coughing, onomatopoeias…) include numerous regions that did not exhibit a significant interaction effect in the current study, such as the bilateral inferior frontal and precentral gyri, the amygdala, and the thalamus [98]. Similarly, previous studies that have investigated functional connectivity changes associated with voice-specific processes such as recognition of familiar voices [56,64], voice monitoring [99], and passive exposure to voices [100] have found modulations between regions distinct from the ones we observed, mostly constrained to the temporal lobes and inferior frontal gyri. Jointly, these studies suggest that our interaction results are not solely attributable to voice familiarity or other voice-related processes.

## Conclusions

Our findings reveal the engagement of the person identity network during speech perception, extending the neural underpinnings of speech processing beyond the canonical language network. Additionally, we show that one of the neurofunctional mechanisms proposed by previous studies as underpinning the interaction between speech and voice processing, i.e., neural overlap for voice and speech processes, is engaged when perceiving speech from a familiar speaker. These findings contribute to understanding the brain mechanisms that support the use of voice priors during speech perception.

## Supporting information

S1 Appendix Original Familiarization text and translation. File additionally contains the statements employed to ensure participant attended to the Familiarization.
(DOCX)

S2 Appendix ROI figures and comparison of ROI coordinates with previous studies. (DOCX)

S3 Appendix Main effect results’ figures and interaction results’ figures in representative participants. (DOCX)

S4 Appendix Comparison of the coordinates of results obtained at the whole-brain level with previous studies. (DOCX)
