# Supplementary material for: Perceiving speech from a familiar speaker engages the person identity network
Source: PLoS One. 2025 May 14;20(5):e0322927. doi: 10.1371/journal.pone.0322927 (PMC12077772; doi:10.1371/journal.pone.0322927)
Supplement: S1 Appendix — File additionally contains the statements employed to ensure participant attended to the Familiarization. (DOCX) [file pone.0322927.s001.docx]

**Appendix 1**

Text S1. Original Familiarization text recorded by both speakers featured in the experimental procedure.

¡Hola! Soy Ana. Ya que me has estado escuchando durante todo este tiempo, y que me vas a oír un poco más antes de que haya acabado este experimento, me parece que lo mínimo que podría hacer es explicarte algunas cosas sobre mí misma. Presta atención, porque después tendrás que contestar unas preguntas. Tengo 18 años y hace poco me mudé a un piso en el barrio de Gracia, en el centro de Barcelona. Curiosamente, ayer una amiga me dijo que, a pesar de ser el barrio más pequeño de toda Barcelona, es el segundo más poblado. Sea como sea, me gusta mucho vivir en él. Disfruto muchísimo paseándome entre sus callejuelas. Me da la impresión que cada día descubro un rinconcito nuevo. Ya había venido un par de veces a Barcelona, principalmente con excursiones del colegio y del instituto, pero hasta ahora solo conocía las “atracciones turísticas”, como la casa Batlló o la Sagrada Familia. No digo que después de las semanas que llevo viviendo aquí sea mucho más que una turista. Como ya he dicho, no hay día que no descubra algo nuevo. Pero sí puedo decir que conozco un restaurante de sushi cerca de casa que hace ofertas los lunes y que, ese mismo día, un cine llamado en honor a un famoso compositor de ópera italiano ofrece entradas a precio reducido para la última sesión de la tarde. ¡Poco a poco voy acumulando conocimientos del local! La razón por la cual me he mudado a Barcelona es muy simple; he venido a cursar mis estudios universitarios. Actualmente estoy en el primer año de biología. Inicialmente mi intención era especializarme en botánica. Siempre me han encantado las plantas y cuidar de ellas. Pero la verdad es que no estoy segura si realmente me gustan tanto como para dedicar mi futura vida profesional a ellas. En lo poco que llevamos de carrera me he entusiasmado con otros campos de la biología, como la ornitología y la micología. Espero que durante el trascurso de la carrera se me aclaren las ideas. En todo caso, esta ha sido mi primera mudanza. Hasta ahora no me había ni cambiado de casa. Me impresionó la cantidad de cajas que fueron necesarias para mover todas mis cosas. No me había dado cuenta hasta ahora de la cantidad de ropa, libros y cosas que he ido acumulando a lo largo de mis 18 años. Lo que más espacio ocupó fueron, sin duda, mis WarHammer. Quizás no sepas de que se trata; es un juego de mesa centrado en la estrategia. Cada jugador tiene sus propias figuras que, previamente, ha pintado cuidadosamente. Llevo jugando desde los 13 años y claro, he acumulado muchas miniaturas. Me daba mucha pena dejarlas juntando polvo en la casa de mis padres, así que me las he traído conmigo. De haber estado mi hermano mayor todavía viviendo en la casa de mis padres habría dejado algunas con él. Pero ahora está estudiando en Madrid. También es un fanático de WarHammer, de hecho, fue él quien me introdujo. En todo caso, ahora que me conoces un poco más, podemos seguir con el experimento.

Text S2. English translation of the Familiarization.

Hello! I'm Ana. Since you've been listening to me all this time, and you'll be hearing more from me before this experiment is over, I think the least I could do is explain a few things about myself. Pay attention because you'll have to answer some questions later. I'm 18 years old, and I recently moved to an apartment in the Gracia neighborhood, in the center of Barcelona. Interestingly, a friend told me yesterday that despite being the smallest neighborhood in all of Barcelona, it's the second most populous. Be that as it may, I really enjoy living here. I love strolling through its narrow streets. I get the impression that every day I discover a new little corner. I had visited Barcelona a couple of times before, mainly on school trips, but until now, I only knew the "tourist attractions" like Casa Batlló or the Sagrada Familia. I'm not saying that after the weeks I've been living here, I'm much more than a tourist. As I mentioned, there's not a day that goes by without me discovering something new. But I can say that now I know a sushi restaurant near my house that has discounts on Mondays, and on the same day, a cinema named after a famous Italian opera composer offers reduced-price tickets for the last evening showing. Little by little, I'm accumulating knowledge about the area!

The reason I moved to Barcelona is very simple; I've come here to pursue my university studies. Currently, I'm in my first year of biology. Initially, my intention was to specialize in botany. I've always loved plants and taking care of them. But the truth is, I'm not sure if I really love them enough to dedicate my future professional life to them. In the short time we've been in the program, I've become enthusiastic about other fields of biology, such as ornithology and mycology. I hope that my ideas will become clearer as I progress in my studies. In any case, this was my first move. Until now, I hadn't even changed houses. I was impressed by the number of boxes it took to move all my stuff. I hadn't realized until now how much clothing, books, and things I had accumulated over my 18 years. What took up the most space was undoubtedly my WarHammers. You might not know what that is; it's a tabletop strategy game. Each player has their own figures that they've carefully painted beforehand. I've been playing since I was 13, and of course, I've collected many miniatures. I was really sad to leave them gathering dust at my parents' house, so I brought them with me. If my older brother had still been living at my parents' house, I would have left some with him. But now he's studying in Madrid. He's also a WarHammer fanatic; in fact, he's the one who introduced me. In any case, now that you know a little more about me, we can continue with the experiment.

Table S1. Questions that participants were required to answer (True/ False) after the presentation of the Familiarization.

| Original text | English translation |
| --- | --- |
| Ana estudia Bellas Artes. | Ana studies Fine Arts. |
| Ana es una aficionada de WarHammer. | Anna is a WarHammer enthusiast. |
| Ana tiene un hermano mayor. | Ana has an older brother. |
| Ana estudia Biología. | Ana studies Biology. |
| Ana se ha mudado hace poco a Burgos. | Ana has recently moved to Burgos. |
