# Supplementary material for: Perceiving speech from a familiar speaker engages the person identity network
Source: PLoS One. 2025 May 14;20(5):e0322927. doi: 10.1371/journal.pone.0322927 (PMC12077772; doi:10.1371/journal.pone.0322927)
Supplement: S2 Appendix — (DOCX) [file pone.0322927.s002.docx]

**Appendix 2**

Figure S1. Clusters defined in this study as voice sensitive and speech sensitive ROIs via an independent functional localizer.


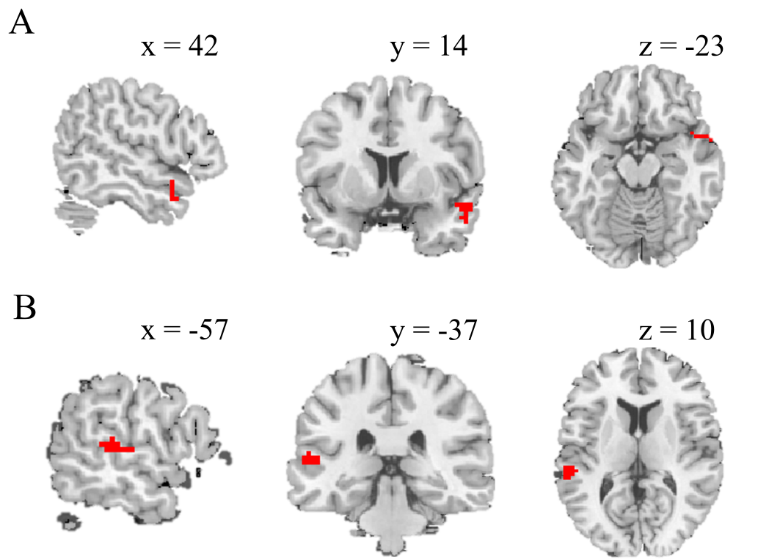


ROIs were defined as all contiguous voxels responsive at *p* < .05 uncorrected located in an anatomical position in line with the literature (see Tables S1 and S2, presented below, for a comparison between these ROIs and those defined by previous studies). A cluster situated in the right temporal pole which extended into the right anterior superior temporal sulcus was defined as the voice-sensitive ROI (MNI coordinates: 42, 14, -23; k = 24 voxels) (panel A). The speech-sensitive ROI was defined as a cluster situated in the left posterior STS (MNI coordinates: -57, -31, 10; k = 33 voxels) (panel B). Anatomical position was determined using the Neuromorphometrics atlas implemented in SPM12.

Table S2. Voice sensitive regions reported by fMRI studies and Euclidean distance to local maxima obtained in the present study. Euclidean distance between local maxima of the present study and previous studies was calculated with the Seed-based *d* Mapping coordinates utility ([www.sdmproject.com/utilities](http://www.sdmproject.com/utilities)). When necessary, Talairach coordinates were converted to MNI space with the Brett transform as implemented in the same web utility. Studies have been organized in ascending order in accordance with the calculated Euclidean distance, with studies that reported peak maxima closer to the local maxima of the present study. To facilitate locating the coordinates referenced here, the terminology of the experimental conditions employed by the referenced studies is reproduced verbatim here.

| Study | MNI local maxima (x,y,z) | Euclidean distance (mm) |
| --- | --- | --- |
| *Present study* | 42, 14, -23 | NA |
| *Andics et al., 2010, n = 25*  Identity Sensitivity | 48, 18, -28 | 8.77 |
| *von Kriegstein et al., 2003, n = 14 **  Voice Task > Sentence Task  Voice Task > Speech Envelope Noise Task | 48, 7, -20  54,13, -16  57, 10, -23 | 9.69  13.96  15.52 |
| *von Kriegstein et al., 2005, n = 19 **  Voice recognition > Verbal Content Recognition  Familiar Speaker > Nonfamiliar Speaker | 48, 22, -19  51, 17, -34 | 10.77  14.52 |
| *Nakamura et al., 2001, n = 9 **  F – V  (F – V) + (F – S) | 32, 14, -29  30, 14, -34 | 11.66  16.27 |
| *Blank et al., 2014, meta-analysis of 64 studies (healthy participants)*  Recognition of famous person identities  Conjunction analysis: Personally familiar and famous person-identity recognition  Recognition of personally familiar person identities | 44, 20, -34  51, 15, -32  53, 11, -33  28, 10, -28 | 12.68  12.76  15.16  15.39 |
| *Imaizumi et al., 1997, n = 6*  Speaker Identification Task | 50, 23, -14 | 15.03 |
| *Latinus et al., 2011, n = 16*  Voice > Non-voice | 57, 5, -14 | 19.67 |

* Studies included in Blank et al., 2014 meta-analysis

Table S3. Speech sensitive regions reported by fMRI studies and Euclidean distance to local maxima obtained in the present study. Euclidean distance between local maxima of the present study and previous studies and conversion from Talairach coordinates to MNI space was conducted with the Seed-based *d* Mapping coordinates utility ([www.sdmproject.com/utilities](http://www.sdmproject.com/utilities)), as reported in supplementary Table 1. Also as in supplementary Table 1, studies have been organized in ascending order in accordance with the Euclidean distance between the reported peak maxima and our own. The terminology of the experimental conditions of the referenced studies is reproduced here.

| Study | MNI local maxima (x,y,z) | Euclidean distance (mm) |
| --- | --- | --- |
| *Present study* | -57, -31, 10 | NA |
| *Hodgson et al., 2021, meta-analysis of 82 phonology experiments and 86 semantics experiments*  Phonology > Semantics  Phonology activation likelihood | -58, -23, 8  -41, -35, 14  -62, -24, 4  -56, -48, 8  -40, -34, 16  -60, -16, -2  -62, -32, 6 | 3.74  19.33  10.48  17.14  18.27  19.44  295.21 |
| *Turkltaub et al., 2010, meta-analysis of 23 experiments*  Speech > Nonspeech | -58, -27, 3 | 8.12 |
| *Scott et al., 2000, n = 8*  Phonetic information (Speech + Noise Vocoded Speech + Spectrally Rotated Speech) - Spectrally Rotated Noise Vocoded Speech)  Intelligibility (Speech + Noise Vocoded Speech) – (Spectrally Rotated Speech + Spectrally Rotated Noise Vocoded Speech) | -64, -38, 0  -60, -4, -10  -54, +6, -16  -56, -12, -12 | 14.07  33.73  29.08  45.32 |
