# Supplementary material for: Perceiving speech from a familiar speaker engages the person identity network
Source: PLoS One. 2025 May 14;20(5):e0322927. doi: 10.1371/journal.pone.0322927 (PMC12077772; doi:10.1371/journal.pone.0322927)
Supplement: S3 Appendix — (DOCX) [file pone.0322927.s003.docx]

**Appendix 3**


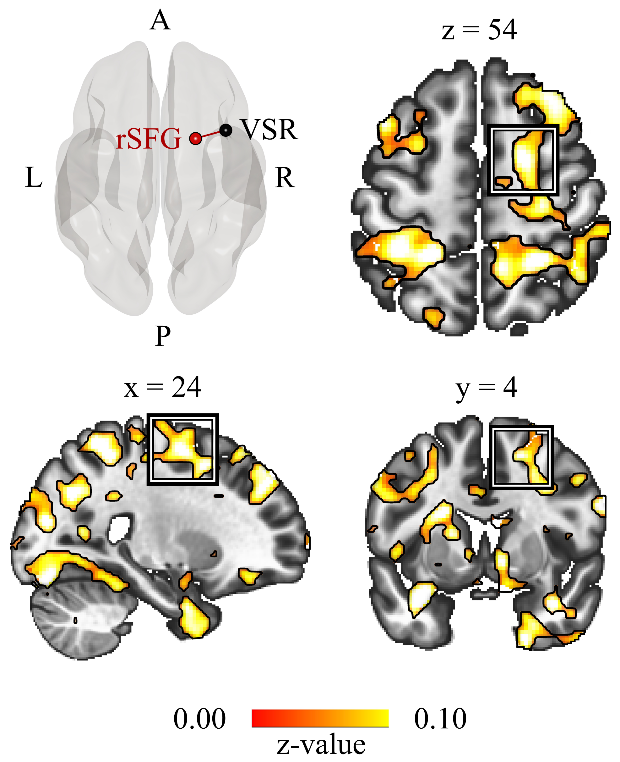


**Fig S2. Functional connectivity interaction contrast in a representative participant.** Glass brain shows the approximate location of the voxel that exhibited the strongest change in functional connectivity strength of the right Superior Frontal Gyrus (rSFG) and Voice Sensitive Region (VSR) in the interaction contrast analysis at the group level. A = anterior. R = right. P = posterior. L = left. Anatomical brain map is overlayed with the seed-based whole-brain functional connectivity map of the interaction contrast of a representative subject. Location of the cluster that is significant at the group level is indicated by a box. Map depicts Fisher z-transformed Pearson correlation coefficients at a threshold of z > .05.


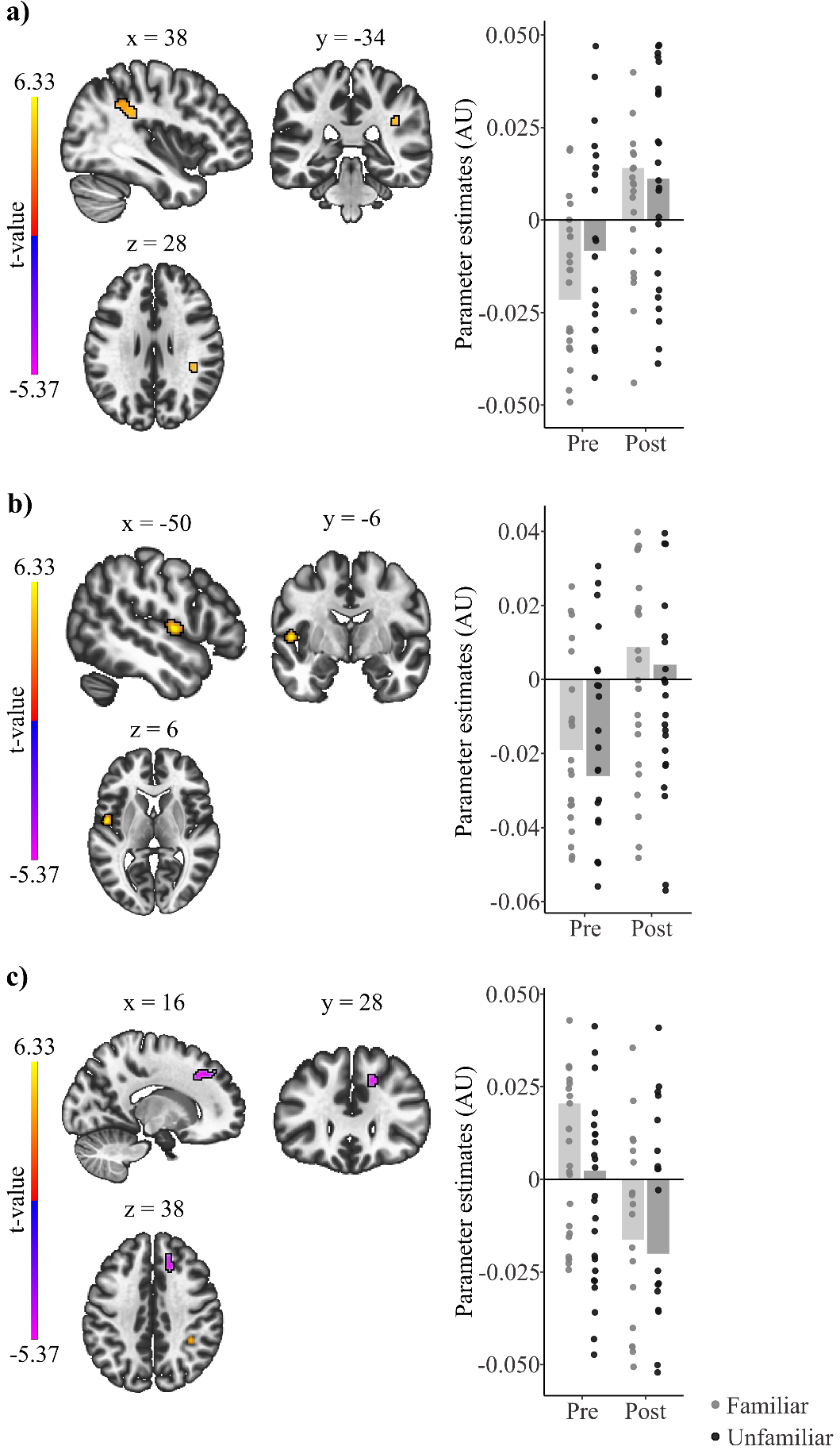


**Fig S3. Results of the seed-to-whole brain functional connectivity analyses of the main effect of run.** Seed was the independently localized voice sensitive region located in the right temporal pole. Significant clusters were in the right supramarginal gyrus (a), left central opercular cortex (b), and right frontal pole (c). Bar-plots (AU: Arbitrary Units) display mean effect sizes associated with each condition for each significant cluster. Participant-specific values for each condition and cluster are represented by the scatterplots overlayed with the respective bar plots. Note that the bar plot legend included in panel C is applicable to all presented bar plots. Results were considered significant at a voxel-wise threshold of p < .001 and cluster-wise threshold of p < .05 FDR-corrected.


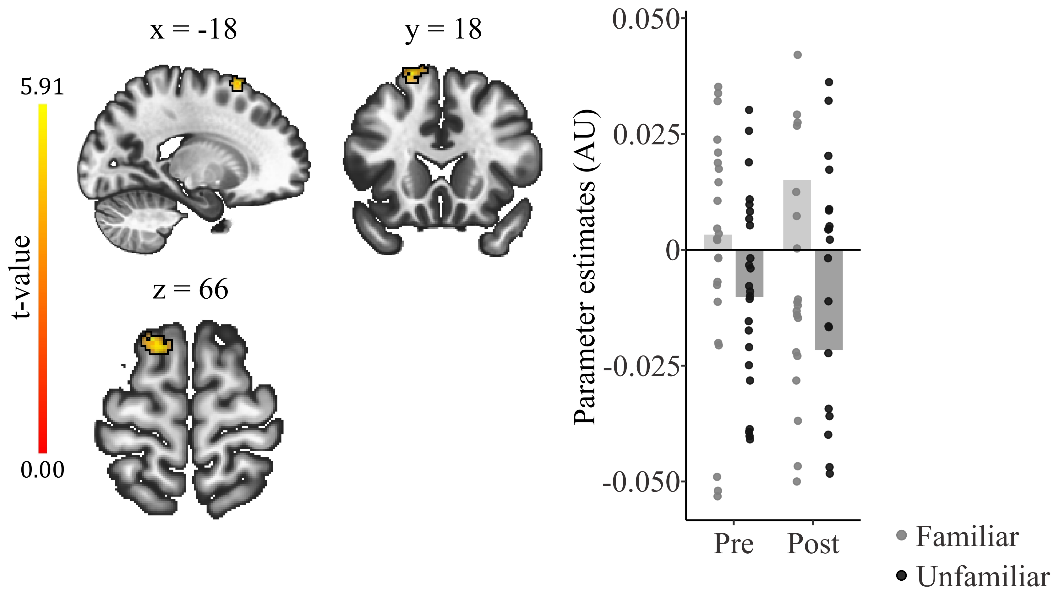


**Fig S4. Results of the seed-to-whole brain functional connectivity analyses of the main effect of speaker.** Seed was the independently localized voice sensitive region located in the right temporal pole. Significant cluster was located in the left superior frontal gyrus. Bar-plots (AU: Arbitrary Units) display mean effect sizes associated with each condition for the significant cluster. Participant-specific values for each condition are represented by the overlayed scatterplots. Results were considered significant at a voxel-wise threshold of p < .001 and cluster-wise threshold of p < .05 FDR-corrected.


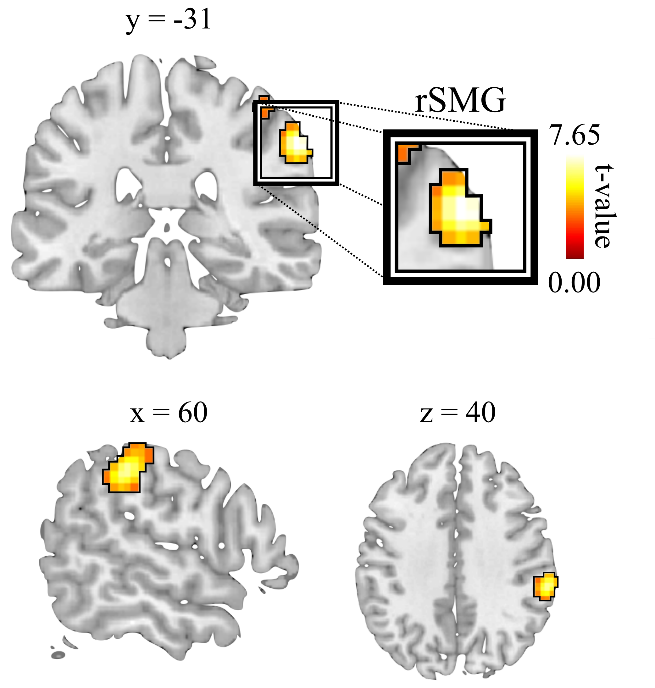


**Fig S5**. **Whole-brain** **BOLD activity interaction contrast in a representative participant.** Result of the interaction contrast in a representative participant shows the engagement of the right supramarginal gyrus. This first-level activation map is thresholded at *p* < .001 uncorrected and intended for display purposes only.


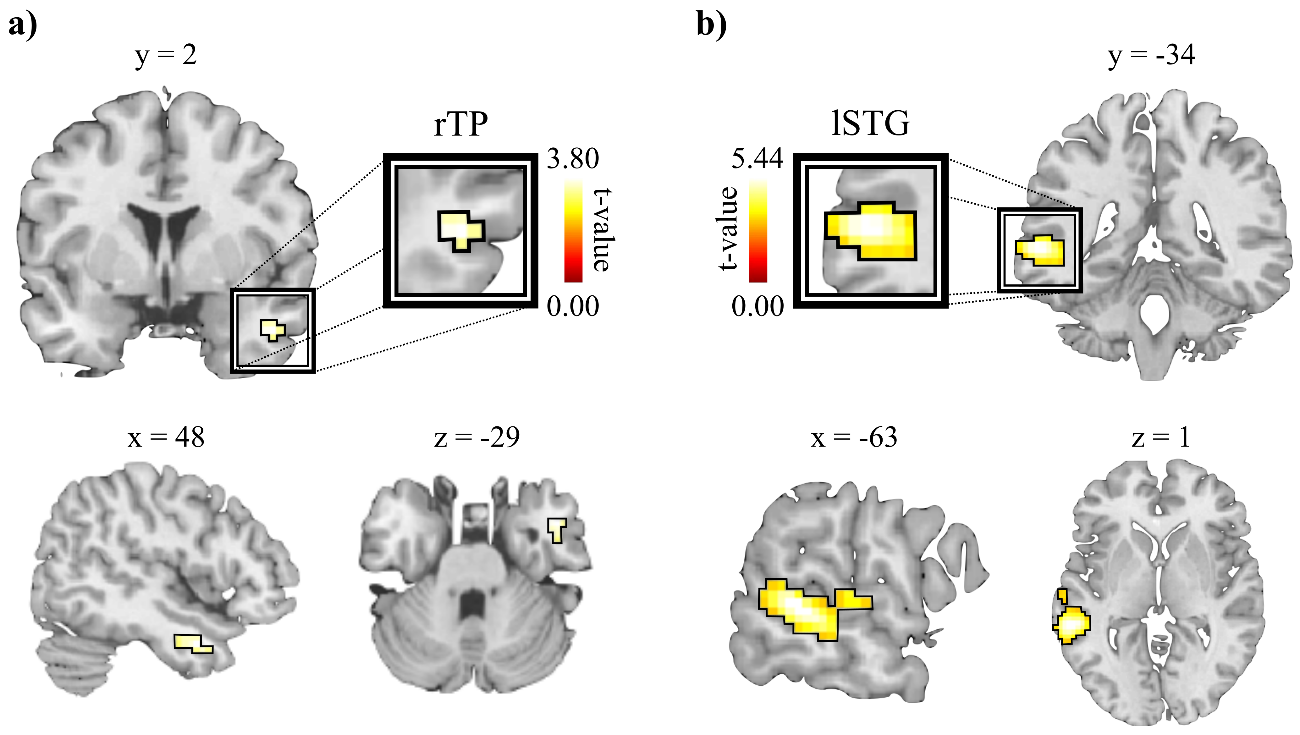


**Fig S6. Representative participant’s results of the interaction contrast analysis of activity in the regions identified as ROIs.** Representative participant’s changes in BOLD activity in the right Temporal Pole (rTP; a) and left Superior Temporal Gyrus (lSTG; b), the regions independently identified as voice-sensitive and speech sensitive, respectively, in the present study. These first-level activation maps have been thresholded at p < .001 uncorrected at the whole brain level and are solely intended for display purposes.


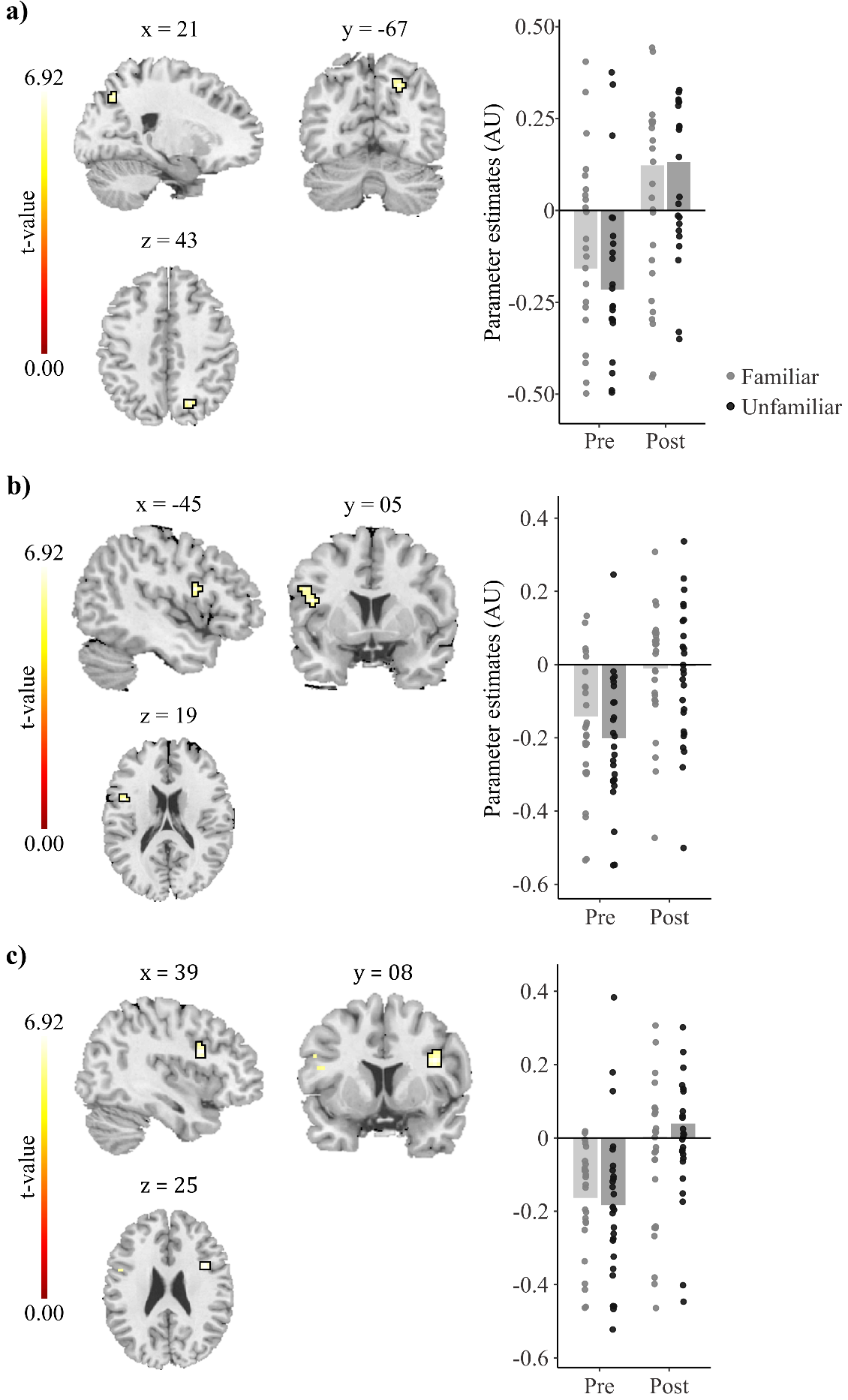


**Fig S7. Main effect of run in BOLD activity at the whole brain level.** Analysis revealed a main effect of run (i.e., Post > Pre) in the right superior parietal lobule (a), left inferior frontal gyrus (b), and right inferior frontal gyrus (c). Bar plots show the parameter estimate, i.e., first eigenvariate, obtained from the peak voxel of each cluster in each condition. Participant-specific values for each condition are represented by the scatterplots overlayed with the respective conditions. Note that the legend of the bar plot presented in panel A is common to all bar plots. Results were considered significant at p < .05 FWE-corrected at the whole brain level (*).
