# Supplementary material for: Perceiving speech from a familiar speaker engages the person identity network
Source: PLoS One. 2025 May 14;20(5):e0322927. doi: 10.1371/journal.pone.0322927 (PMC12077772; doi:10.1371/journal.pone.0322927)
Supplement: S4 Appendix — (DOCX) [file pone.0322927.s004.docx]

**Appendix 4**

Table S4. Location of the right superior frontal gyrus reported by fMRI studies investigating verbal working memory (i.e., the cognitive ability that allows us to retain and mentally manipulate linguistic information) and Euclidean distance to local maxima obtained in the present study. Euclidean distance between local maxima of the present study and previous studies was calculated with the Seed-based *d* Mapping coordinates utility ([www.sdmproject.com/utilities](http://www.sdmproject.com/utilities)). When necessary, Talairach coordinates were converted to MNI space with the Brett transform as implemented in the same web utility. Studies have been organized in ascending order in accordance with the calculated Euclidean distance, with studies that reported peak maxima closer to the local maxima of the present study listed first. To facilitate transparency, the terminology that the referenced studies employ to refer to the contrasts is reproduced verbatim.

| Study | MNI local maxima (x,y,z) | Euclidean distance (mm) | |
| --- | --- | --- | --- |
| *Present study* | 24, 4, 54 | NA | |
|  | Verbal working memory | | |
| *Emch et al., 2019, meta-analysis of 42 studies ** |  | |  |
| Load-effect meta-analysis results | 22, 14, 58 | | 10.39 |
|  | 8, 32, 48 | | 32.80 |
| *Marvel et al., 2010, n = 16 ** |  | | |
| Positive Activations | 4, 18, 53 | 24.43 | |
|  | 6, 68, 16 | 76.57 | |
| *Strand et al., 2008, n = 12* |  |  | |
| [ISI WM]-[S1 Ctrl] | 0, -3, 66 | 27.73 | |
|  | -6, 3, 57 | 30.16 | |
|  | -6, 15, 51 | 32.09 | |
| [S1 WM]-[S1 Ctrl] | -6, 0, 60 | 30.85 | |
|  | -3, 9, 69 | 31.28 | |
|  | -3, 0, 72 | 32.69 | |
| [S2 WM]-[S1 Ctrl] | -3, 39, 27 | 51.79 | |

*Study included in the Emch et al., 2019 meta-analysis.

Table S5. Location of the right supramarginal gyrus reported by previous studies investigating person identity recognition, phoneme predictability, and speaker-specific phoneme enunciation sensitivity. Table includes the Euclidean distance to the local maxima obtained in the present study. Euclidean distance between local maxima of the present study and previous studies was calculated with the Seed-based *d* Mapping coordinates utility ([www.sdmproject.com/utilities](http://www.sdmproject.com/utilities)). When necessary, Talairach coordinates were converted to MNI space with the Brett transform as implemented in the same web utility (i.e., Seed-based *d* Mapping). Studies have been organized in ascending order in accordance with the calculated Euclidean distance and the function attributed to the SMG, with studies and functions that reported peak maxima closer to the local maxima of the present study listed first. The terminology that the referenced study employs to refer to the contrasts associated with the referenced coordinates is reproduced verbatim.

| Study | MNI local maxima (x,y,z) | Euclidean distance (mm) |
| --- | --- | --- |
| *Present study* | 54, -37, 31 | NA |
|  | Person Identity Recognition | |
| *Kruse et al., 2016, n = 14* |  | |
| Decoding familiar other from unfamiliar other (FAM/UNFAM) | 57, -42, 33 | 6.16 |
| *Awwad Shiekh Hasan et al., 2016, n = 5* |  |  |
| Voice -> Face | 66, -26, 28 | 16.55 |
| *Bestelmeyer & Mühl, 2022, n =* 40 |  |  |
| (B) Voice Test (covariate effect) | 66, -22, 23 | 20.80 |
|  | Phoneme predictability | |
| *Vaden et al., 2010, n = 17* |  | |
| Repetition-Suppression | 63, -27, 33 | 13.60 |
|  | Speaker-specific phoneme enunciation sensitivity | |
| *Myers & Theodore, 2017, n = 17* |  | |
| Typical > Atypical | 44, -58, 24 | 24.28 |
